# Examining the External Antecedents of Innovative Work Behavior: The Role of Government Support for Talent Policy

**DOI:** 10.3390/ijerph18031213

**Published:** 2021-01-29

**Authors:** Zaisheng Zhang, Meng Liu, Qing Yang

**Affiliations:** 1 College of Management and Economics, Tianjin University, Tianjin 300072, China; zhangzs@tju.edu.cn; 2 School of Accounting, Shanxi University of Finance and Economics, Taiyuan 030012, China; 20171063@sxufe.edu.cn

**Keywords:** sustainable innovation performance, innovative work behavior, government support, high-performance work system

## Abstract

The innovative work behavior (IWB) or creativity of employees is regarded as the key to the sustainable innovation performance of an organization. In the field of human resource management (HRM), the relationship between an organization’s high-performance work system (HPWS) and IWB has been studied extensively. However, the current understanding of organizational external antecedents is limited. Our paper focuses on an extra-organizational government support factor, government support for talent policy (GSTP). Similar to HRM policies within an organization, GSTP also has an ability–motivation–opportunity framework that may influence the IWB of employees. We integrate the resource dependence theory, institutional theory, and theory of planned behavior (TPB) to propose a theoretical model of the mechanism of GSTP influence on IWB. Using a structural equation modeling approach, we empirically verify the hypotheses in a survey dataset of HRM practitioners in 152 technology-based enterprises in China. The results indicate that the external antecedent, GSTP, positively influences the innovative attitude, subjective norm, and perceived behavioral control of HRM practitioners in the organization. Innovative attitude and perceived behavioral control completely mediated the relationship between GSTP and innovative intention. Moreover, there is a distal indirect effect between GSTP and IWB. The paper contributes to filling a gap in the innovation policy literature. In practice, both HPWS and individual employees should be concerned about the possible role of GSTP.

## 1. Introduction

The innovative work behavior (IWB) or creativity of employees is regarded as the core of organizational innovation [1,2,3]. Therefore, more studies are needed to improve the understanding of the relevant factors of high-level individual innovative behavior [4].

Whether an individual employee adopts a positive IWB will be influenced by the organization’s high-performance work system (HPWS), which is generally considered to be a human resource management (HRM) system that includes selective recruitment, high-level training and development, competitive compensation and benefits, and extensive participation and information sharing [5,6]. Moreover, the micro perspective is increasingly used by scholars to discover and clarify the influence mechanism of this relationship [7]. From this micro perspective, employees’ attitudes, beliefs, and opinions are regarded as important mediating means to establish this relationship [8,9]. For example, Ramamoorthy et al. found that incentive compensation can make innovative behavior generate an obligation to promote innovation [10]. Employees’ confidence and emotions will be improved through empowerment, and they will be more willing to put new ideas into practice [11]. Jiang et al. found that different goal dimensions of HPWS can have different impacts on employee and organizational outcomes [12].

What is worth further attention is that the antecedents from outside the organization cannot be ignored, especially the variables at the government and national levels [13]. Blom et al. found that HR policies and practices within organizations are strongly influenced by government factors [14]. At present, governments have used a variety of support tools to promote innovation, especially the Chinese government [15,16]. In terms of human resources, from the 2008 economic crisis to the present, China’s central government and local governments have provided government support for talent policies (GSTP), such as the “Thousand Talents Program”, “The National Science Fund for Distinguished Young Scholars,” etc. [17]. Promoting human resource development and talent innovation are the main objectives of GSTP. With such an important organizational external influence variable in HR, it is important to explore the potential impact of GSTP on the innovative intentions and behaviors of employees within an organization. However, so far, our understanding of the relationship is limited. Moreover, research evidence about the indirect psychological process, that is, whether and how antecedent factors affect individuals’ innovative intention or behavior, needs to be improved further [18].

To address the gap in the literature, we integrate the resource dependence theory, institutional theory, and theory of planned behavior (TPB) theory to construct a theoretical model of the hypothesized relationship between the GSTP and IWB of employees. Based on resource dependency theory, enterprises are constrained by external resources and should, therefore, seek to acquire resources from different sources to reduce their dependence on each one [19,20]. GSTP is provided as an optional resource, and organizations probably try to acquire this external resource to enhance the chance of organizational survival. Based on institutional theory, GSTP from the government will exert a constraining institutional pressure on the organization. Organizations, in return for higher legitimacy, resources, and survival, choose to comply with institutional pressures [21]. As individuals who are affiliated with the organization and actually utilize GSTP resources, employees’ IWBs are inevitably affected by the GSTP. For the transduction pathway of GSTP affecting IWB, the TPB has been widely regarded as contributing to explain the mechanisms affecting innovation intention and behavior. TPB emphasizes the integrated influence of individual attitude, subjective norm, and perceived behavioral control (PBC) on intention and behavior [22]. Therefore, we hypothesize that GSTP further influences innovative intention and behavior by affecting individuals’ innovative attitude, subjective norm, and PBC. We empirically verified the hypotheses through a survey of HRM practitioners from technology-based enterprises in Tianjin, China.

The expected contribution of this paper is both theoretical and empirical. It mainly includes the following three points: first of all, compared to the many innovation policy studies that focus on subsidy policy and intellectual property policy, our study focuses on GSTP. We found that GSTP and HPWS have similar HRM objectives in the ability–motivation–opportunity domain. This paper contributes to the literature in the field of human resources for government innovation policy research. Our study will provide a new perspective for the follow-up study on the relationship between the constructs. Secondly, based on the resource dependence theory, institutional theory, and TPB, we theoretically determine and discuss the influence mechanism of GSTP on individual employees’ innovative intention and IWB. In other words, we have theoretically explained the “how” and “why” of these relationships. Thirdly, under the extended TPB model, we empirically verify the direct and indirect influence paths of GSTP on HR practitioners’ innovative intention and IWB. The paper explores the psychological process mechanism of external government support to individual IWB in organizations. 

This paper proceeds as follows: In Section 2, we summarize the theoretical background and propose our hypotheses. The data and methods are described in Section 3. In Section 4, the results of the model are calculated and presented. After that, we discuss the results in Section 5. Section 6 gives our conclusions.

## 2. Theoretical Background and Research Hypotheses

### 2.1. Theoretical Background

#### 2.1.1. Government Support for Talent Policy (GSTP)

The government is one of the most important actors influencing organizations and individuals to achieve innovation [23]. Research on government support is extensive [15,24]. While the role of the government in enterprise innovation is controversial, the growing government support for innovation-related activities is undeniable [23,25]. Due to the unique cultural and institutional background of China, the Chinese government, compared to many other countries, has strong power to allocate resources [26]. Many resources are used by the government to support the innovative activities of enterprises. Since the financial crisis of 2008, China’s central government and local governments have provided many GSTPs [17].

A distinctive feature of strategic HRM research is the emphasis on HRM system (or HPWS) rather than individual HR practices as drivers of individual and organizational performance [12,27]. Employee performance is a more direct result of HRM systems than organizational performance [28]. One benefit of focusing on broadly considered employee performance is that employees are expected to contribute to organizational effectiveness regardless of the organization’s strategic goals [5]. Appelbaum et al. argue that HRM systems aimed at maximizing employee performance can be considered a combination of three dimensions (aimed at improving employee abilities, motivation, and opportunities to contribute, as AMO dimensions for short) [12,29]. Lepak et al. further conceptualized three domains of HR policies and practices based on the AMO framework [27]. In keeping with these studies, many studies have divided organizational HRM policies and practices into three domains. Jiang et al. systematically summarized the HRM system components and argue that the immediate goal of the HRM system at the highest level is to achieve the desired employee performance. To achieve this superordinate goal, the goals of the HRM policy area can then be divided into three domains: policies in the ability domain (e.g., selection, training), policies in the motivation domain (e.g., performance management, rewards), and policies in the opportunity domain (e.g., job engagement, job design) [5,24].

The delineation of HRM policies based on the AMO framework informs our study. Although the HRM policies within the organization may differ from GSTP in terms of the scope of employees covered and the operational mechanism, they are similar as far as the goal framework is concerned. At the highest level, both are similarly focused on improving employees’/talents’ performance, and to achieve this goal, the HRM policy and GSTP can be categorized into three-goal dimensions. Therefore, based on the AMO framework, we summarize the goal domains of GSTP, using Tianjin city as an example. As shown in Table 1, GSTP has similar HRM goals compared to HRM policies in organizations. The predominance of policies in the ability and motivation domains is also similar to the distribution of HRM policies within organizations [14]. According to the table, both the enterprise and the individual level may be targeted for support. At the individual level, the direct beneficiaries of the GSTP may be talents (especially R&D talents) and HRM practitioners in an organization. Our study focuses on HRM practitioners in organizations, a group that is certainly well informed about GSTP. The organization’s HRM practitioners are the mediator between the external GSTP and the organization. In contrast to some talents who may benefit from only one domain of GSTP, HRM practitioners can utilize GSTP resources in each goal domain, at the organization level or at the individual level, to accomplish specific HRM practices, as part of their job duties, such as recruiting, training, motivating, and encouraging innovation.

#### 2.1.2. Theory of Planned Behavior (TPB)

TPB has been widely used to analyze the intention and influencing factors of human behavior [30,31]. Fishbein and Ajzen first proposed the theory of rational behavior [32], from which Ajzen extended and developed TPB [22]. According to TPB, the intention of human behavior is the main factor that determines executive behavior; and an individual’s attitude, subjective norm, and perceived behavioral control (PBC) are assumed to be the direct antecedents of behavioral intention. Specifically, attitude is the positive, negative, or indifferent perception of something [22]. When an individual decides to execute a certain behavior or not, the perceived external pressure is the subjective norm. For example, when an individual decides to smoke or drink alcohol, he/she needs to consider the feelings of the people around him/her [33]. The perception of the difficulty of executive behavior is PBC. Ajzen found that attitude, subjective norm, and PBC had positive effects on behavioral intention, and behavioral intention further positively predicted executive behavior [34]. This theoretical model has been used by many scholars to study intention and behavior, and it has been further extended to include a wide range of psychological, individual, and social policy factors [35].

### 2.2. Research Hypotheses

Intention determines an individual’s willingness and plan to try something. Personal intention is considered the most direct and important factor to predict individuals’ future behavior [36]. Hence, intention can be called the best tool to predict individual behavior. In the TPB model, the more favorable the antecedents of intention, attitude, subjective norm, and PBC are, the more willing an individual is to attempt a specific behavior [37]. Many previous studies have shown that behavioral intention can predict individual behavior in different fields [38]. Innovation is not an exception, because intention will lead to a strong desire and effort to engage in innovative behavior [39]. Through individuals’ intention of innovative behavior, we can better understand the process of an individual’s attempt to engage in innovative behavior. Therefore, we propose Hypothesis 1.

**Hypothesis** **1** **(H1).** *Innovative intention is positively related to IWB.*

The ultimate result of an individual’s attitude is action [34]. Attitude involves the assessment of the overall performance of an individual’s behavior. Ajzen argues that if a person evaluates his/her behavior and believes that it will lead to positive results, then he/she will have a positive attitude toward the expected behavior [34]. Hence, if a person has a positive evaluation of behavior, then he/she will have a positive attitude toward the behavior. In the context of innovation, individual attitudes play a dominant role in the legitimization, adoption, and even implementation of new initiatives [40]. Individuals’ attitudes may affect their intention, behavior, and organization’s strategies and activities. Therefore, we propose the second hypothesis.

**Hypothesis** **2** **(H2).** *Innovative attitude is positively related to innovative intention.*

Ajzen proposed that subjective norm is a type of perception of external expectation and pressure [22]. Subjective norm is closely related to the acceptance of behavior by the reference. When an individual engages in activity and thinks that he/she has met the expectations of others (especially the influential reference), he/she reduces social pressure [41]. Such pressure comes from different factors in the external environment. Previous studies have shown that subjective norm plays an important role in the influence of behavioral intention [42]. Some scholars also found that the influence of subjective norm is not always significant, which may be due to measurement error or an undetected indirect effect [43]. Thus, the relationship between them is more likely to be positive. Therefore, we propose the third hypothesis.

**Hypothesis** **3** **(H3).** *Subjective norm is positively related to innovative intention.*

When an individual thinks that he/she has the ability to achieve the expected behavior, he/she is more likely to succeed [41]. An individual’s willingness to engage in a specific behavior is affected by PBC, especially when the individual thinks that the behavior can be under his/her control. PBC has two key dimensions [34]. The first dimension is self-efficacy. An individual with high self-efficacy is likely to take advantage of opportunities and to be willing to take risks [44,45]. The second dimension is controllability, which is the individual’s perception of facilitating or limiting behavior. When an individual perceives that he/she has the necessary resources and opportunities for innovation, he/she may have a higher intention of innovative behavior [46]. The literature also finds that, even when an individual perceives inadequate control, he/she nonetheless assesses the likelihood of obtaining the necessary resources elsewhere to increase the probability of engaging in innovative development [47]. Therefore, we propose Hypotheses 4 and 5.

**Hypothesis** **4** **(H4).** *PBC is positively related to innovative intention.*

**Hypothesis** **5** **(H5).** *PBC is positively related to IWB.*

Resource dependency theory describes an organization as an open system that is constrained by resources [48]. Some studies argue that firms should strive to obtain resources from different sources to reduce their dependence on each resource [19,20]. The resources provided by the government may increase the chances of survival, especially for Small and medium-sized enterprises (SMEs) [49]. Positive change in attitudes toward innovation has also been suggested as a key goal of government support [50,51]. In the context of innovation, Gary Chapman argues that an innovation-oriented attitude can be divided into three dimensions: support for innovation, risk tolerance, and openness to external knowledge [52]. Following Chapman’s view, we know that the government tries to encourage employees to achieve innovative performance through the GSTP. In this process, the GSTP may provide HR practitioners with direct experience (e.g., innovation vouchers) or intuitive assessment information (e.g., government-sponsored selection programs) for their managerial practices. These intuitive experiences and information can enhance their ability to innovate and increase their confidence to successfully manage or directly perform innovative activities in the future [53]. The additional resources provided by the GSTP will stimulate firms to experiment with high-risk management innovations in their HR activities. HRM practitioners may also gain direct risk-related experience in the process. These experiences with risky decisions can influence their risk appetite and increase their risk tolerance [54]. Finally, Lichtenthaler and Ernst suggest that external (positive) knowledge and experiences affect attitude change [55]. Government support for HR business exchanges, professional training, etc., can provide HRM practitioners with professional information, knowledge, and opportunities to interact with peers outside the organization. Based on the above discussion, we propose the following hypothesis:

**Hypothesis** **6** **(H6).** *GSTP is positively related to innovative attitude.*

Institutional theory suggests that the institutional environment can greatly influence organizational development, even more so than pressures from the marketplace [56]. Examples of institutional environments include laws, government regulations, government guidelines, and social culture, etc. [57]. The institutional environment can exert a constraining influence on the organization, called isomorphism, and isomorphic pressures are of three types: coercive, normative, and mimetic. These institutional forces can influence organizational structure, climate, and behavior [56]. Different interests in the institutional environment can have a strong influence on organizational and individual behavior [58]. Liang et al. found that institutional pressure was significant in the assimilation of external institutional pressures on the use of enterprise resource planning (ERP) systems, which positively influenced top management’s beliefs and, consequently, their engagement behavior [59]. Wang argues that as IT innovation evolves and the level of institutionalization increases, the institutional environment is likely to become more accepting of innovation [60]. According to institutional theory and related research, GSTPs come from government agencies and have institutional pressures that expect organizations to innovate. Organizations generally choose to comply with institutional pressures in return for greater legitimacy, resources, and viability [61]. Although HRM practitioners are individual agents, they are also governed by the actions of the organizations they represent. HRM practitioners may perceive the pressures and expectations of GSTP. The subjective norm is a perception of external expectations and pressures. Therefore, we propose Hypothesis 7.

**Hypothesis** **7** **(H7).** *GSTP is positively related to subjective norm.*

Intellectual capital within an organization (including HRM practitioners) can be described as the most important intangible resource. In terms of how to efficiently leverage an organization’s resource endowment, Hillman et al. suggest that combining resource dependency theory with a resource-based view may be particularly fruitful [62]. PBC is an individual employee’s understanding of the degree of difficulty of the desired target behavior [34]. Venkatesh et al. argue that controllability is an individual employee’s perception of external conditions that can be influenced by facilitating conditions such as resources, technology, etc. [63]. Alalwan et al. further argued that facilitating conditions include external government support [64]. Nasri and Charfeddine argue that an individual’s desired behaviors are likely to be perceived as more feasible when he/she perceives high-level government support [65]. GSTP provides resources to the organization and talents through business training, peer networking events, etc. HRM practitioners may gain knowledge and skills through these activities. These opportunities can facilitate individual employees to consciously practice skills and activities that prepare them for creativity [66]. So, GSTP may also directly increase the HRM practitioner’s control over management and practice innovation.

**Hypothesis** **8** **(H8).** *GSTP is positively related to PBC.*

We inferred an indirect effect of GSTP on innovation intentions through its effects on innovative attitude, subjective norm, and PBC. Three organizational and behavioral theories support this relationship, including resource dependence theory [48], institutional theory [56], and TPB [22]. Under the resource dependency theory and institutional theory perspectives, organizations will seek the help of GSTP to obtain more survival opportunities and sources of resources to reduce unexpected uncertainty. As an intra-organizational contact group for GSTP, the innovative attitude, subjective norm, and PBC of HRM practitioners can be positively influenced by GSTP. Under the extended TPB model, innovative attitude, subjective norm, and PBCs further influence the innovative intentions of HRM practitioners. Therefore, we hypothesize an indirect relationship between the above concepts:

**Hypothesis** **9a** **(H9a).** *Innovative attitude positively mediates the relationship between GSTP and innovative intention.*

**Hypothesis** **9b** **(H9b).** *Subjective norm positively mediates the relationship between GSTP and innovative intention.*

**Hypothesis** **9c** **(H9c).** *PBC positively mediates the relationship between GSTP and innovative intention.*

**Figure 1:**
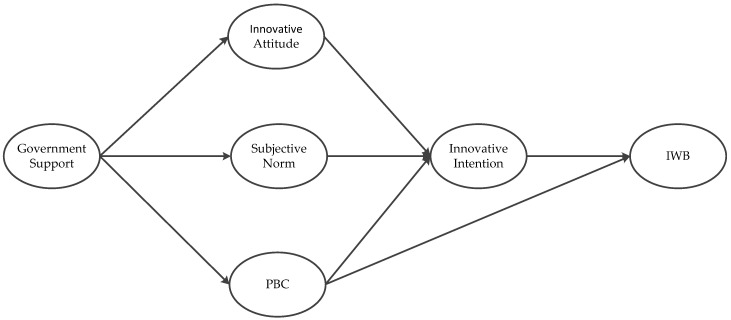
The hypothetical framework.

Figure 1 illustrates the theoretical relationship between the above concepts.

## 3. Materials and Methods

### 3.1. Measurement

The key variables in the study were measured using a seven-point Likert scale (1 = strongly disagree; 7 = strongly agree). The measurement scales were all derived from well-established scales that have been widely cited by many studies. The expressions of the measurement items were appropriately adapted to Chinese respondents. All constructs, item content, standard factor loadings, and Cronbach’s α values are reported in supplementary material Table S1.

#### 3.1.1. Government Support for Talent Policy

The measurement of this latent variable is adapted from Li and Atuahene-Gima’s four-item measurement scale on government support [24], which is combined with the AMO framework. The scale includes the perception of government support for selective recruitment, training, performance, and encouraging innovation.

#### 3.1.2. Innovative Attitude

The scale is mainly extracted from the scale of Chapman and Hewitt [52], which refers to a large number of previous scales [1,40]. It measures individuals’ innovative attitude from three aspects: support for innovation, risk tolerance, and openness to external knowledge.

#### 3.1.3. Subjective Norm

Measures of subjective norms were derived from an earlier scale by Ajzen [22], with appropriate adaptations by Carmeli and Schaubroeck [67] to fit the context of innovation. Our measurement items were extracted from Carmeli and Schaubroeck’s scale. 

#### 3.1.4. Perceived Behavior Control

The measure of this latent variable was earlier derived from Ajzen’s scale and was adapted for different purposes in the study [67]. Our scale was directly adapted from Taylor and Todd [68].

#### 3.1.5. Innovative Intention

Choi measured innovative intention based on Fishbein and Ajzen’s Behavioral Intention Scale [69]. The scale only had two items at first, but Li et al. and other scholars enriched the scale [70]. We cite two measurements from Choi and one measurement from Li et al.

#### 3.1.6. Innovative Work Behavior

The IWB scale developed by Janssen has been cited by many researchers [2]. The scale consists of three parts: the generation of ideas, the promotion of ideas, and the realization of ideas, with a total of nine topics. We cited seven items from Janssen’s scale.

#### 3.1.7. Control Variables

Based on previous studies, we selected gender, education level, age, and work experience as control confounding variables [71,72]. Madrid et al. believed that a higher number of working years can increase individual confidence about innovative ideas [71]. Carmeli and Spreitzer proposed controlling the variables of education level and age [72] because they believed these two variables would have positive and negative effects on innovation, respectively.

### 3.2. Sample Selection and Data Collection

In comparison with the previous fragmented talent policies, the Tianjin Municipal Government systematically integrated and implemented the GSTP in May 2018. Emerging industries such as information technology, advanced manufacturing, new energy and new materials, environmental protection, biology and medicine are the main target industries for GSTP. Enterprises in these industries are knowledge-intensive and high value-added, and therefore have a relatively high percentage of talents. Based on the objectives, the data of this paper were collected from HRM practitioners of technology-based enterprises in Tianjin, China.

The survey is divided into an email survey and a field survey. Using the list of technology-based SMEs and the list of emerging industry enterprises authorized by Tianjin City in 2020, we sent an email to 1718 technology-based enterprises to invite them to participate in the research. The email contained the specific requirements of the survey and a link to the questionnaire. To encourage respondents to participate, monetary rewards and e-mail reminders were used. In the end, we received 109 responses to the email survey. In the field survey part, we sent questionnaires to select technology-based enterprises through the human resource recruitment market. We invited these enterprises to answer the questionnaire if they had not already participated in the online survey. We distributed 246 questionnaires and recovered 64. Finally, after excluding 21 invalid questionnaires, 152 valid questionnaires were obtained. The samples were collected in two ways, so the issue of sampling bias needs to be considered. Referring to Fleming and Bowden [73], the results of the chi-square test and contingency table analysis indicated that there were no significant differences between the two samples in the distribution of demographic variables or the structure of key variables. The issue of sampling bias was not significant. Descriptive statistical information of the respondents being shown in supplementary material Table S2.

### 3.3. Common Method Bias Test

Common method biases (CMB) may arise in the self-report method [74]. Therefore, we refer to the relevant recommendations of Podsakoff et al. [74]. First, we tried to avoid or reduce CMB through the following methods. In principle, each enterprise only invites one HRM practitioner to fill in the form to avoid a common background. Respondents fill them in anonymously to protect the confidentiality of interviewees, and we switched the question order to control the retrieval cues prompted by the question context. Second, we use Harman’s one-factor test. According to relevant recommendations, when a single factor exceeds a 50% variance, the CMB problem is considered serious [75]. Our test results show that the first principal component accounts for a 25.046% variance. This means that the first factor is far from explaining most of the variance. Therefore, CMB is unlikely to be a major issue in our research.

## 4. Results

Following the recommendations of Anderson and Gerbing [76], we adopted a two-step procedure involving CFA and structural equation modeling (SEM) to analyze the data.

### 4.1. Measurement Modeling Analysis

We conducted a series analysis of the measurement models mainly using the Mplus 7.0 software (Table 2 shows the results). Finally, the Cronbach’s alpha values > 0.7, indicating that the questionnaire’s measurement passed the internal consistency reliability test. The standardized factor loadings for each item were all greater than 0.6, the combined reliability (CR) values were all greater than 0.7, and the average variance extracted (AVE) values were overwhelmingly greater than 0.45, indicating that our measured variables were generally well convergent [77]. All square roots of AVE values were greater than the Pearson’s correlation coefficients between the latent variables, indicating that the measured variables have good discriminant validity [78]. Through the above tests, we know that our measurement model has good reliability, convergent validity, and discriminant validity.

To further test the convergent and discriminant validity of the measurement model, we compared the hypothesized six-factor model with other possible alternative models. The CFA results are presented in Table 3 and support the discriminant validity of the six-factor model. The indicators of the six-factor model are in line with the recommended values (χ^2^ values minimum, and 1<χ^2^ /df < 3; root mean square error of approximation (RMSEA) = 0.041 < 0.08; comparative fit index (CFI) = 0.951 > 0.9; Tucker–Lewis index (TLI) = 0.943 > 0.9; standardized root mean square residual (SRMR) = 0.062 < 0.08) [76]. As expected, the six-factor measurement model is reasonable, which provides evidence for satisfactory discriminant validity.

### 4.2. Hypothesis Testing

The SEM technique provided by Mplus 7.0 software was used to test structural models containing mediators. Compared to Baron and Kenny’s method, the SEM approach is more appropriate for our study [79]. The process described by Bagozzi and Edwards is followed [80]. We compared the hypothesized fully mediated model 2 with other potential alternative structural models.

**Figure 2:**
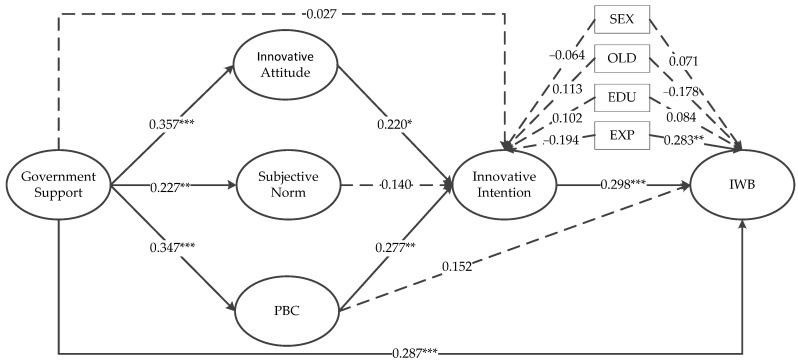
Path coefficients for the final structural model. * *p* < 0.10; ** *p* < 0.05; *** *p* < 0.01. Solid arrows indicated the significant paths. Dotted arrows indicated the insignificant paths.

As shown in Table 4, model 4 is our final structural model. Model 1 has a very good overall fit, but this is not the ultimate goal of our study. Model 2 is our hypothesized fully mediated model, and it does not differ significantly from the partially mediated model 3 in the values of χ^2^, RMSEA, CFI, and TLI. This indicates that GSTP and innovative intention are more likely to be fully mediated relationships. The value of χ^2^ for model 4 decreases, the RMSEA and SRMR values become smaller, and the CFI and TLI values increase closer to 1. Therefore, based on these comparisons between potential structural models, we retained alternative model 4 as the final structural model. The standardized path coefficients for this model are shown in Figure 2.

As shown in Figure 2, most of the hypotheses were verified. Both innovative attitude (β = 0.220, *p* < 0.100) and PBC (β = 0.277, *p* < 0.050) had a positive effect on innovative intention. H2 and H4 are supported. H3 regarding subjective norm (β = 0.140, *p* > 0.100) was not supported. Innovative intention (β = 0.298, *p* < 0.010) had a positive effect on IWB, and H1 was supported. However, PBC (β = 0.152, *p* > 0.100) did not directly affect IWB, and H5 was not supported. The hypothesis that GSTP has a positive impact on the innovative intention of HRM practitioners within an organization was empirically verified. GSTP had a positive impact on innovative attitude (β = 0.357, *p* < 0.010), subjective norm (β = 0.227, *p* < 0.050), and PBC (β = 0.347, *p* < 0.010). H6, H7, and H8 are supported. For possible indirect paths in the structural model, we used the MODEL INDIRECT command of the MPLUS 7.0 software to test for direct and indirect effects. This command was used to obtain standard errors and significance levels for indirect effects via the default delta method [81]. The results showed that the two paths of innovative attitude (β = 0.078, *p* < 0.100) and PBC (β = 0.096, *p* < 0.050) fully mediated the relationship of GSTP on innovative intention. H9a and H9c are supported, while H9b is not supported. Moreover, GSTP has distal indirect effects on IWB through PBC, innovative intention (β = 0.029, *p* < 0.100). This distal indirect effect was partial, because GSTP had a direct positive relationship on IWB (β = 0.287, *p* < 0.010). In addition, the positive effect of work experience on IWB was significant at the 5% level, and none of the remaining cases regarding the control variables were significant. Finally, we consider the robustness tests in supplementary material 3.

We further test the importance of indirect effects by using the bootstrap approach. For tests of mediating and moderating effects, the bootstrap approach is considered more reliable and can produce results with higher accuracy than normal theoretical tests (e.g., Sobel test) [82]. We continued the mediation effects analysis, based on a sample of 5000 bias-corrected bootstrap programs with 95% confidence intervals (CIs). According to the results shown in Table 5, innovative attitude (B = 0.081, SE = 0.064, 95% CI = [0.002, 0.279]) and PBC (B = 0.099, SE = 0.063, 95% CI = [0.013, 0.286]) significantly mediated the positive relationship of GSTP on innovative intention. Beyond the normal hypothesized relationship, the distal indirect effect of GSTP on IWB was also significant for the distal indirect path through PBC, innovative intention (B = 0.020, SE = 0.079, 95% CI = [0.001, 0.309]). The three indirect effects were shown to be supported by the bias-corrected bootstrap analysis.

## 5. Discussion

Our study focuses on extra-organizational government support factors in the field of HR. We test an extended TPB model that describes the mechanistic impact of GSTP on innovative attitude, subjective norm, PBC, innovative intention, and IWB. The model can be believed to provide many valuable results.

### 5.1. Theoretical Significance

First, our paper contributes to the literature on innovation policy from a human resources perspective. We draw a summary of GSTP based on the AMO goals framework. The summary of GSTP enriches the talent policy component of Huang and Wang’s study of China’s innovation policy system [23,25]. Our study extends Jiang’s application of the classification of HR policies within organizations [5]. Furthermore, we find that GSTP influences the innovative intention and behaviors of HRM practitioners within organizations. Therefore, we strongly call attention to the GSTP, an external variable that may influence organizational and individual innovation. Efficient development of human resources is considered an important key in the background of the general aging of the population and the upgrading of the economy [83]. Similar to industrial and financial policies, GSTP is becoming an important tool for governments to support enterprise innovation in both emerging and developed economies [25,84]. Our study will provide a new perspective for the follow-up study of the relationship between the constructs.

Secondly, we theoretically and empirically determine and discuss the influence mechanism of GSTP on individual employees’ innovative intention and IWB. Based on the resource dependence theory, Bouchard et al. argue that corporations should strive to acquire resources from different sources to reduce their dependence on each of them [19,20]. Chapman finds that government support for innovation vouchers has a positive impact on managers’ innovative attitude. Our study validates Chapman’s argument and finds that HRM practitioners respond positively to government support [52]. This may be the result of HRM practitioners’ evaluation of the new information brought about by government support and their determination of a new information matrix. Our study partially supports Wang’s view that government support is accompanied by institutional pressures that reinforce the expectation that corporations need to innovate [60]. In addition, the PBC path is equally significant, which empirically enriches Hillman’s assertion that resource dependency theory and resource-based theory are integrated [62].

Third, by extending the TPB model, we empirically verify the direct and indirect psychological mechanism pathways of GSTP on HR practitioners’ innovative intention and IWB. The results show that there is a fully mediated relationship between GSTP and innovative intention. This paper may be an extension of Chapman’s view [52]. Government support not only affects the manager’s innovative attitude but also indirectly affects innovative intention through the path of innovative attitude. Besides, we are more in agreement with Nasri’s findings on the relationship between government support and PBC [65]. Furthermore, we find that there is a distal indirect effect between GSTP and IWB, through the path of PBC and innovative intention. The distal indirect relationship has been less discussed in previous studies. Our study of the mechanisms of psychological processes is comprehensive and insightful.

Finally, as in many parts of the world, SMEs in China are a very important part of the domestic economy [85], but less is known about the impacts of how to enhance the innovation capacity of SMEs [86]. SMEs must be creative and diligent due to the difficulty of gaining more from economies of scale [87]. Development around employee knowledge becomes critical for organizations to achieve competitive advantage [88], i.e., ultimately by promoting IWB of employees in order to enhance performance. Our empirical results with the main sample of technology-based SMEs find that GSTP can positively influence innovative attitude, subjective norm, and PBC, as well as further influence IWB. This adds to the empirical evidence of the relationship between government support and SMEs. In other words, government support affects behavior not only at the organizational level, but also at the individual level within the organization, especially for SMEs that are relatively lacking in resources. GSTP may also have a resource or signaling effect on employees to enhance their innovative attitudes, subjective norms, and PBC, based on our empirical results.

### 5.2. Practical Significance

Our evidence suggests that GSTP can influence IWB at the employee level. Hillman et al. compare resource dependency theory with resource base theory and argue that there is a need to consider how an organization identifies its resource needs and how to obtain these valuable resources [62]. Therefore, the GSTP needs to further match the resource needs of intangible resources and can include improving individual PBC as one of the matching target points. GSTP is one of the most effective tools to promote positive attitude change in individuals. We agree with Chapman’s suggestion to call attention to the issue of attitudes in policy implementation [52]. Governments need to consider the possible impact on individual attitudes when formulating talent policies.

The organization’s HPWS needs to strengthen its interaction with government support. We have summarized the GSTP based on the AMO framework and empirically verified its positive impact on IWB. HPWS needs to compete for and take advantage of the resources provided by the government for recruitment, training, innovation subsidies, etc. HPWS may increase the selectivity of resources to complete the practice of recruiting, training, rewards, and opportunities.

As individuals within an organization, especially HRM practitioners, they should take into account resources from outside the organization and leverage the GSTP for recruiting, training, and rewarding, etc. Hana, Sun, and Wang argued, based on the job resource–job demand model (J-D model) [89], that, although organizational HPWS provides individuals with a wide range of job resources, it also increases the demand for individual jobs. Under the J-D model, the target pressure, rigidity, and traceability of government job demands may be relatively easy to accomplish compared to corporate. Individual employees may have greater autonomy and control over resources to experiment with innovative behavior.

### 5.3. Limitations and Future Research

Although this paper makes significant contributions to the relevant literature, we need to be cautious about the findings, because our paper has deficiencies and limitations. First, this cross-sectional study can only draw positive or negative effects, but a credible causal inference cannot be made. The causal inference needs further experimental design and longitudinal data for in-depth study. Second, the self-reported questionnaire may lead to the artificial expansion of the effect [67]. Nonetheless, we have taken countermeasures and found that the effect may not be a serious problem. Third, our samples are mainly based on the survey of technology-based SMEs in Tianjin City, China. Therefore, replication studies in other regions and other types of enterprises are needed to improve the accuracy and universality of the conclusions. Third, our study is preliminary and future findings from more types of enterprises and respondents are needed to improve the possible accuracy and generalizability of the findings. The policy instruments of the GSTP may differ across countries and cultures, as we are surveying in the context of East Asia, a region with more powerful governments. The GSTP may be differentially affected by the moderating effects of macro-level variables, such as the regional institutions, and organization-level variables, such as HPWS effectiveness and organizational innovation climate. Our survey respondents were limited to the HRM practitioner group. Other groups, particularly the R&D talent group, may have more complex mechanisms affected by GSTP. Finally, the study should be based on a larger sample size to eliminate possible outliers and increase the robustness of the conclusions.

## 6. Conclusions

A theoretical model is constructed and empirically verified based on the resource dependence theory, institutional theory, and TPB. The results show that GSTP positively influences the innovative intention and IWB of HRM practitioners within an organization. In terms of psychological influence pathways, GSTP can positively and directly influence innovative attitude, subjective norm, and PBC. There is a fully mediated relationship between GSTP and innovative intention, mainly through the innovative attitude and PBC paths, with the subjective normative path being insignificant. In addition, we find that there is a distal indirect effect between GSTP and IWB, through the path of PBC and innovative intention.

## Figures and Tables

**Table 1:** Government support for talent policies (GSTP) in the abilitity-motivation-opportunity framework.

Type	Name	Narrative
Abilities Domain	Recruitment Policy	Job fairs; subsidies or incentives for talent recruitment; relaxation of household registration; incentives for intermediaries, etc.
	Select Policy	Special skill competitions; special honor projects, etc.
	Training Policy	Government subsidies for skills training activities and programs; subsidies for participation in academic exchanges; subsidies and incentives for training platforms, etc.
Motivation and Effort Domain	Performance Management Policy	Promote talent evaluation reform to classified and market-based evaluation (for state-owned enterprises and government departments), etc.
	Compensation Policy	Wage and living allowances, etc.
	Incentives Policy	Science and technology transformation awards; funding for innovation projects; funding for the operation of innovative technology platforms; honorary awards, etc.
Opportunities to Contribute Domain	Involvement Policy	Granting partial authority for the use of research funds; high-level talent symposiums.
	Job Design Policy	None

Note: Based on the Talent Policies of the Tianjin Municipal Government. Source of Reference: http://publicservices.hrss.tj.gov.cn/ecdomain/framework/zcydt/fbbafimfopkibboikmfajfanojgaanpa.jsp?term=421&typetree=421.

**Table 2:** Reliability, convergence validity, and discriminate validity results.

DIM	Item Reliability	Composite Reliability	Convergence Validity	Discriminant Validity					
	STD Loading	CR	AVE	GSTP	ATT	SN	PC	II	IWB
GSTP	0.680~0.715	0.787	0.481	**0.694**	-	-	-	-	-
ATT	0.647~0.787	0.754	0.507	0.295	**0.712**	-	-	-	-
SN	0.604~0.932	0.795	0.572	0.177	0.333	**0.756**	-	-	-
PC	0.640~0.808	0.818	0.530	0.314	0.239	0.383	**0.728**	-	-
II	0.614~0.822	0.744	0.496	0.209	0.263	0.283	0.373	**0.704**	-
IWB	0.616~0.739	0.847	0.442	0.355	0.582	0.189	0.384	0.381	**0.665**

Note: The right side of the table except the diagonal is the Pearson correlation matrix of the variables. Square root of AVE for each construct was shown in the diagonal of the correlation matrix and was bolded. STD Loading: Standardized factor loading. CR: Combined reliability. AVE: Average variance extracted. GSTP: Government support for talent policy. ATT: Innovative attitude. SN: Subjective norm. PBC: Perceived behavior control. II: Innovative intention. IWB: Innovative work behavior.

**Table 3:** Competitive measurement model comparison.

Models	χ^2^	df	Δχ^2^	RMSEA	CFI	TLI	SRMR
One-factor model	859.560	252	563.311	0.126	0.494	0.446	0.118
Two-factor model	750.865	251	454.616	0.114	0.584	0.542	0.117
Three-factor model	641.351	249	345.102	0.102	0.673	0.638	0.114
Four-factor model	536.132	246	239.883	0.088	0.758	0.729	0.097
Five-factor model	418.759	242	122.510	0.069	0.853	0.832	0.076
Six-factor model	296.249	237	-	0.041	0.951	0.943	0.062

Note: *n* = 152, χ^2^: Overall Model Chi-Square Measure; TLI: Tucker–Lewis Index; CFI: Comparative Fit Index; RMSEA: Root Mean Square Error of Approximation; SRMR: Standardized Root Mean Square Residual. Δχ^2^ is a comparison of χ^2^ for all alternative models with the hypothetical six-factor model. One-factor model: all variables are loaded into 1 factor. Two-factor model: GSTP, innovative intention, and IWB combine into 1 factor; innovative attitude, subjective norm, and PBC combine into another factor. Three-factor model: GSTP and innovative intention constitute one factor; IWB is one factor; innovative attitude, subjective norm, and PBC constitute the third factor. Four-factor model: GSTP is one factor; innovative intention is one factor; IWB is one factor; innovative attitude, subjective norm, and PBC constitute the fourth factor. Five-factor model: GSTP is a factor; innovative intention is a factor; IWB is a factor; innovative attitude is a factor; subjective norm and PBC constitute the fifth factor. Six-factor model: GSTP, innovative intention, IWB, innovative attitude, subjective norm, and PBC are all independent factors.

**Table 4:** Competitive structure model fitting index.

Models	χ^2^	df	RMSEA	CFI	TLI	SRMR
Alternative Model 1	275.122	234	0.034	0.958	0.952	0.075
Baseline Model 2	429.532	332	0.044	0.918	0.908	0.091
Alternative Model 3	429.076	331	0.044	0.918	0.908	0.090
Alternative Model 4	421.252	330	0.043	0.924	0.914	0.084

Note: *n* = 152. Alternative Model 1: The standard TPB model. Baseline Model 2: Baseline Model. GSTP is added as an antecedent variable to the standard TPB model. It is hypothesized that innovative attitude, subjective norm, and PBC fully mediate the relationship between GSTP and innovative intention. Alternative Model 3: Partial mediation model. Compared to Baseline Model 2, Alternative Model 3 adds a direct path from GSTP to innovative intention. Alternative Model 4: A partly mediated model including IWB path. Alternative Model 4 adds direct paths between GSTP and IWB based on Alternative Model 3.

**Table 5:** Results of indirect effects test based on the bias-corrected bootstrapping method.

Indirect Paths	B	SE	Bias-Corrected 95% CI	
			Lower Limit	Upper Limit
GSTP → ATT → II	0.081	0.064	0.002	0.279
GSTP → SN → II	0.033	0.034	−0.005	0.151
GSTP → PBC → II	0.099	0.063	0.013	0.286
GSTP → II → IWB	0.006	0.063	−0.099	0.071
GSTP → PBC → IWB	0.038	0.066	−0.032	0.122
GSTP → ATT → II → IWB	0.017	0.027	0.000	0.147
GSTP → SN → II → IWB	0.007	0.008	0.000	0.037
GSTP → PBC → II → IWB	0.020	0.079	0.001	0.309

Note. *n* = 152. B = non-standardized path coefficient. CI = confidence interval. CIs are based on the bias-corrected bootstrapping of 5000 samples. GSTP: Government support for talent policy. ATT: Innovative attitude. SN: Subjective norm. PBC: Perceived behavior control. II: Innovative intention. IWB: Innovative work behavior.

## Supplementary Materials

The following are available online at https://www.mdpi.com/1660-4601/18/3/1213/s1, Table S1: The constructs, item content, std. factor loadings, and Cronbach’s α values, Table S2: Descriptive statistics of the respondents, supplementary material 3: The results of robustness tests.
