# Supplementary material for: Examining the External Antecedents of Innovative Work Behavior: The Role of Government Support for Talent Policy
Source: Int J Environ Res Public Health. 2021 Jan 29;18(3):1213. doi: 10.3390/ijerph18031213 (PMC7908344; doi:10.3390/ijerph18031213)
Supplement: Supplementary file 1 [file ijerph-18-01213-s001.pdf]

Supplementary Material Table S1.

Table S1. The constructs, item content, std. factor loadings, and Cronbach's α values.

Constructs	Measures	Item Content	Std. Loading	Cronbach's α
GSTP	GSTP1	Our company really benefits from the help of GSTP in recruiting and keeping talents.	0.715	0.785
	GSTP2	Our company really benefits from the help of GSTP in the training of our employees.	0.676	
	GSTP3	Our company really benefits from the help of GSTP in motivating talents (compensation for performance).	0.702	
	GSTP4	Our company does benefit from the GSTP in encouraging the talents to innovate.	0.680	
ATT	ATT1	I actively seek innovative ideas and approaches in my work.	0.787	0.747
	ATT2	I try to assist other members in developing new ideas and approaches.	0.695	
	ATT3	I am willing to venture into new ideas and approaches to accomplish my work.	0.647	
SN	SN1	I think my supervisors want me to be creative in my work.	0.695	0.781
	SN2	I think my colleagues would like the new ideas and approaches that I propose at work.	0.932	
	SN3	I think my work team needs me to come up with new ideas and approaches.	0.604	
PC	PC1	When I am faced with the difficult task, I believe I will accomplish the work creatively.	0.700	0.815
	PC2	I believe I have the knowledge, resources, and ability to creatively accomplish work tasks.	0.808	
	PC3	I believe I can control whether I adopt new ideas and new approaches to accomplish tasks.	0.754	
	PC4	I am a strong learner and can quickly absorb new knowledge and skills related to my work tasks.	0.640	
II	II1	I have a strong motivation to propose new and constructive working proposals.	0.660	0.734
	II2	At work, I am willing to actively seek new ideas or solutions to solve problems.	0.822	
	II3	I am willing to proactively share my new ideas or solutions to problems with colleagues.	0.614	
IWB	IWB1	I seek newer approaches, techniques or tools to work.	0.629	0.845
	IWB2	I provide original solutions to problems.	0.673	
	IWB3	I mobilize people around me to support my work ideas.	0.739	
	IWB4	I make key organizational members to be enthusiastic about my work ideas.	0.616	
	IWB5	I put innovative ideas into practice and turn them into useful applications or solutions.	0.665	
	IWB6	I introduce innovative ideas into the work environment in a relatively systematic way.	0.654	
	IWB7	I evaluate the realistic usefulness of my innovative ideas.	0.672	

Note: GSTP: Government support for talent policy. ATT: Innovative attitude. SN: Subjective norm. PBC: Perceived behavior control. II: Innovative intention. IWB: Innovative work behavior.

Supplementary material Table S2.

Table 2. Descriptive statistics of the respondents.

Item	Content	Percent	Item	Content	Percent
SEX	Male	41.45%	EXP	0–5 years	26.97%
	Female	58.55%		6–10 years	28.95%
IND	Other	15.79%		11–15 years	27.63%
	Electronic information technology	21.05%		16–20 years	9.21%
	Biology and Medicine	14.47%		≥20 years	7.24%
	New materials and new energy	10.53%	YEAR	0–5 years	22.37%
	High-tech service	10.53%		6–10 years	34.21%
	Resource and environmental technology	11.84%		11–15 years	26.32%
	Aerospace industry	1.32%		16–20 years	9.21%
	Advanced manufacturing	14.47%		≥20 years	7.89%
POS	HRM supervisor	31.58%	REV	<0.01 billion yuan	27.63%
	HRM manager	33.55%		0.01–0.019 billion yuan	21.71%
	Other department manager	5.26%		0.02–0.099 billion yuan	25.00%
	Senior executive	13.16%		0.10–0.39 billion yuan	15.79%
	Other	16.45%		≥0.40 billion yuan	9.87%
OLD	≤25 years old	11.84%	PEO	<100 people	45.39%
	26–35 years old	44.74%		100–299 people	31.58%
	36–45 years old	30.26%		300–499 people	14.47%
	46–55 years old	12.50%		500–999 people	5.26%
	>55 years old	0.66%		≥1000 people	3.29%
EDU	Under college	1.32%			
	College degree	17.76%			
	Bachelor degree	71.05%			
	Master degree and above	9.87%			

Note: n = 152. SEX: Sex of Respondents. IND: Industries. POS: Position. OLD: Age of respondents. EDU: Education level. EXP: Working Experience. YEAR: Age of the company. REV: Operating revenue. PEO: Number of people.

Supplementary Material 3.

We test the robustness of the results by adding control variables and changing the independent variables. We test the robustness of the results by adding control variables and changing the independent variables. The issue of omitted variables is of most concern. HRM practitioners may be more likely to develop IWB in enterprises that are highly innovative. but the innovative capability is not directly observed. Since we invited only one HRM practitioner for a company in principle. We therefore considered adding firm-level control variables while changing the GSTP to a 0–1 dummy variable (Dgstp = 0, if GSTP value ≤ 16). As companies age, organizations rely on their original development paths to develop organizational inertia [1], which can hinder the absorption of new knowledge and limit innovation [2]. Regarding organizational size, large corporations have greater R&D capabilities and risk tolerance than small firms. Large corporations are also considered to have a greater absorptive capacity [3], but may also lack the flexibility to acquire and assimilate new external knowledge [2]. Based on the above studies, we selected revenue, number of employees, firm age, and the industry as firm-level control variables to proxy for unobserved variables such as possible innovative capacity. The regression results are shown in Table S3, the first column adds firm-level control variables, and in the second column we add a dummy variable of Dgstp, which is significant at the 10% level. HRM practitioners who obtained the GSTP were 1.550 times the odds of attempting IWB than those who did not. In the third column, we consume more degrees of freedom by adding the industry variable. Although Dgstp becomes insignificant (p = 0.109), it is very close to the 10% level of significance. Thus, it is reasonable to assume that the effect of GSTP on IWB is generally robust.

Table S3. The results of robustness tests.

Variables	IWB		
	**Model 1**	**Model 2**	**Model 3**
sex	0.178	0.411	0.180
	(0.265)	(0.605)	(0.263)
old	−0.233	−0.260	−0.509
	(−0.403)	(−0.453)	(−0.869)
edu	1.080 *	1.014 *	0.938
	(1.877)	(1.770)	(1.613)
exp	0.461	0.567	0.609
	(1.061)	(1.299)	(1.369)
rev	0.748 **	0.687 **	0.773 **
	(2.183)	(2.007)	(2.215)
peo	−0.185	−0.243	−0.396
	(−0.434)	(−0.573)	(−0.891)
year	−0.400	−0.393	−0.146
	(−1.192)	(−1.179)	(−0.404)
D.gstp		1.550 *	1.534
		(1.691)	(1.614)
1.ind			1.911 *
			(1.789)
2.ind			0.486
			(0.412)
3.ind			0.672
			(0.513)
4.ind			0.382
			(0.298)
5.ind			2.158
			(1.653)
6.ind			−2.231
			(−0.774)
7.ind			−0.576
			(−0.487)
_cons	32.007 ***	30.819 ***	30.431 ***
	(14.123)	(13.065)	(12.241)
N	152	152	152
Adj. R-sq	0.024	0.036	0.050

Note: t statistics in parentheses. * $p < 0.1$, ** $p < 0.05$, *** $p < 0.01$.

References

1. Xie, Z.; Li, J. Exporting and innovating among emerging market firms: The moderating role of institutional development. *J. Int. Bus. Stud.* **2018**, *49*, 222–245.
2. Duan, Y.; Wang, W.; Zhou, W. The multiple mediation effect of absorptive capacity on the organizational slack and innovation performance of high-tech manufacturing firms: Evidence from Chinese firms. *Int. J. Prod. Econ.* **2020**, 107754.
3. Kulkarni, S.S. A framework and model for absorptive capacity in a dynamic multi-firm environment. *Int. J. Prod. Econ.* **2015**, *167*, 50–62.
